# 

**Published:** 1848-04

**Authors:** 


### CONTENTS

OF NO. IV.

### ORIGINAL COMMUNICATIONS.

### ESSAYS AND CASES.

ART. I.—Ether Inhalation in Convulsions. By RICHARD C. WYATT, M.D., of Shelby county, Tennessee. . . . . . . . . . . . . . .277
ART. II.—Datura Stramonium an Emmenagogue. By B. F. JONES, M.D., of St. Louis county, Missouri. . . . . . . . . . . .282
ART. III.—Reports of Cases of Disease; with Remarks. By JOHN DAWSON, M.D., of Jamestown, Ohio. . . . . . . . . . .285
ART. IV.—Remarks on the Use of the Letheon; with Cases. By J. F. SANFORD, M.D., of Farmington, Iowa . . . . . . . .302
ART. V.—A Case of Convulsions terminating fatally, caused by Ascaris Lumbricoides; Appearances Post Mortem. By WILLIAM T. WINSOR, M.D., of Lexington, Mo. . . . . . . . .308

### REVIEWS.

ART. VI.—Females and their Diseases; a Series of Letters to his Class. By CHARLES D. MEIGS, M.D., Professor of Midwifery and the Diseases of Women and Children in the Jefferson Medical College at Philadelphia; Member of the American Medical Association; of the American Philosophical Society, and one of the Council; Fellow of the College of Physicians of Philadelphia; one of the Physicians to the Lying-in Department of the Pennsylvania Hospital, etc. Philadelphia: Lea and Blanchard. 8vo. pp. 670. 1848. . . . . . . . . . . . . . . . . . . . . . . . . . . . . . . . . .310
ART. VII.—A Practical Treatise on the Causes, Symptoms and Treatment of Spermatorrhœa. By M. LALLEMAND, formerly Professor of Clinical Surgery at the University of Montpellier; Member of the Royal Academy of Medicine of Paris, etc. Translated and edited by HENRY J. MCDOUGALL, Member of the Royal College of Surgeons of England; Fellow of the Royal Medical and Chirurgical Society; and formerly House Surgeon to the University College Hospital. Philadelphia: Lea and Blanchard. 1848 ........ 330
Art. VIII.—Elements of the Principles and Practice of Midwifery. By David H. Tucker, M.D., Professor of the Principles and Practice of Medicine, and formerly of Obstetrics and the Diseases of Women and Children, in the Franklin Medical College of Philadelphia. With numerous Illustrations. Philadelphia: Lindsay and Blakiston: 1848. 412 pp. 12mo ........ 335
Art. IX.—The United States Farriery, and Zoological History of Horses, Cattle, Sheep and Hogs; with Engravings; connected with the Therapeutical Illustrations of Medicine. Compiled from the most approved Authors, by Buell Eastman, M.D. Cincinnati: C. Cropper and Son. 1848 ........ 336

SELECTIONS FROM AMERICAN AND FOREIGN JOURNALS.

Treatment of Asiatic Cholera ........ 337
Epilepsy Successfully Treated with Nitrate of Silver, etc ........ 345
Death from the Inhalation of Chloroform, in Cincinnati ........ 348
Cure of Spina Bifida by Injection of Tincture of Iodine ........ 352
Death of Professor Dieffenbach ........ 354
Application of Nitrate of Silver to the Larynx in Croup ........ 355
A Case of Spontaneous Evolution of the Fœtus ........ 359

ORIGINAL INTELLIGENCE.

Progress of the Use of Chloroform ........ 361
Kentucky Institution for the Blind ........ 363
Sanitary Survey ........ 364
Ranking's Half-Yearly Abstract ........ 365
Munificent Contribution to Medical Education ........ 366
Allopathy versus Homœopathy ........ 367
American Medical Association ........ 367

